# Addressing Challenges of Opportunistic Computed Tomography Bone Mineral Density Analysis

**DOI:** 10.3390/diagnostics13152572

**Published:** 2023-08-02

**Authors:** Kirsten N. Bott, Bryn E. Matheson, Ainsley C. J. Smith, Justin J. Tse, Steven K. Boyd, Sarah L. Manske

**Affiliations:** 1 Department of Radiology, University of Calgary, Calgary, AB T2N 1N4, Canada; kirsten.bott@ucalgary.ca (K.N.B.); skboyd@ucalgary.ca (S.K.B.); 2 McCaig Institute for Bone and Joint Health, University of Calgary, Calgary, AB T2N 4Z6, Canada; 3 Department of Biomedical Engineering, Schulich School of Engineering, University of Calgary, Calgary, AB T2N 1N4, Canada

**Keywords:** computed tomography, internal BMD calibration, opportunistic analysis

## Abstract

Computed tomography (CT) offers advanced biomedical imaging of the body and is broadly utilized for clinical diagnosis. Traditionally, clinical CT scans have not been used for volumetric bone mineral density (vBMD) assessment; however, computational advances can now leverage clinically obtained CT data for the secondary analysis of bone, known as opportunistic CT analysis. Initial applications focused on using clinically acquired CT scans for secondary osteoporosis screening, but opportunistic CT analysis can also be applied to answer research questions related to vBMD changes in response to various disease states. There are several considerations for opportunistic CT analysis, including scan acquisition, contrast enhancement, the internal calibration technique, and bone segmentation, but there remains no consensus on applying these methods. These factors may influence vBMD measures and therefore the robustness of the opportunistic CT analysis. Further research and standardization efforts are needed to establish a consensus and optimize the application of opportunistic CT analysis for accurate and reliable assessment of vBMD in clinical and research settings. This review summarizes the current state of opportunistic CT analysis, highlighting its potential and addressing the associated challenges.

## 1. Introduction

In Canada, from 2003 to 2019, the number of computed tomography (CT) scans performed increased by 96% [1]. As diagnostic imaging continues to be an important clinical tool, the number of CT scans is projected to steadily rise. There remains a wealth of unused data that is captured in a CT scan outside of the initial diagnostic purpose [2] that may be leveraged for secondary analysis. As the population continues to age and healthcare costs increase [3], optimizing and repurposing clinical data for disease screening, diagnostics improvements, and facilitating research is a top priority.

CT has long been used in the research setting to study physiological and pathological changes in bone. Advances in hardware and computational analysis have extended our ability to secondarily analyze clinical CT scans beyond the scope of the initial diagnostic purpose. However, it is difficult to reliably compare Hounsfield units (HU) across different CT scanners and scan protocols given that HU are influenced by X-ray energy, photon flux, positioning within the scanner, spatial resolution, beam hardening, and other artefacts [4]. Additionally, research methods often require quantification metrics, which cannot be acquired without image calibration. While calibration phantoms remain the gold standard, the development of phantomless internal calibration techniques has improved the reliability of secondary CT analyses, without the need for a traditional calibration phantom within the scan field of view [5,6]. However, current phantomless internal calibration techniques are limited in their robustness, emphasizing the need to develop standardized phantomless internal calibration methods to effectively address these limitations.

Given the suitability of CT imaging for the assessment of bone and the established association between low bone mineral density (BMD) and increased risk of fracture [7], opportunistic CT analysis has largely focused on osteoporosis screening by measuring volumetric BMD (vBMD). It is a cost-effective preliminary screening tool that can be used to determine if an individual should undergo a dual energy X-ray absorptiometry (DXA) scan for the clinical diagnosis of osteoporosis [8,9,10]. Although DXA is the clinical standard for diagnosis, CT is particularly valuable given that it provides a three-dimensional view of the skeleton unlike the two-dimensional areal BMD (aBMD) from DXA scans or traditional X-rays. It is important to note that CT vBMD typically measures the vertebral body, which differs from DXA aBMD that measures the whole vertebra including the processes. An advantage of CT imaging is the ability to segment bone including anatomical sites (e.g., vertebral body, femoral neck) or bone compartments (e.g., trabecular, cortical bone). Moreover, CT grayscale values are proportional to vBMD [11] and provide a high contrast between bone and soft tissues, unlike magnetic resonance imaging (MRI) techniques. Although outside the scope of this review, CT scans can be further used for bone strength estimations using finite element modelling and/or texture analysis [12,13]. Previous reviews have summarized the use of opportunistic CT analysis specifically for the clinical assessment of osteoporosis [14,15,16,17,18]; however, measurements of vBMD from secondary CT analysis can also facilitate musculoskeletal research.

Secondary analysis of clinically acquired CT scans can be analyzed to address research questions such as the effects of various diseases and conditions (e.g., cancer, diabetes, Crohn’s disease) as well as longitudinal changes in vBMD over the lifespan, without requiring further scan resources or radiation exposure to the individual. However, there are technical considerations associated with emerging techniques for secondary analysis of CT images that are the focus of this review. As musculoskeletal research continues to evolve towards multi-centre and longitudinal studies, standardizing opportunistic vBMD analysis from a variety of CT scanning methods and protocols is essential. Some of the primary concerns for opportunistic CT vBMD analysis are calibration accuracy, the influence of contrast agents routinely used in clinical CT scans, methods for bone segmentation, and adapting vBMD calibration to new CT technologies. The purpose of this review is to summarize the current state of opportunistic CT analysis and highlight these key challenges.

## 2. Opportunistic CT vBMD Analysis

**Figure 1:**
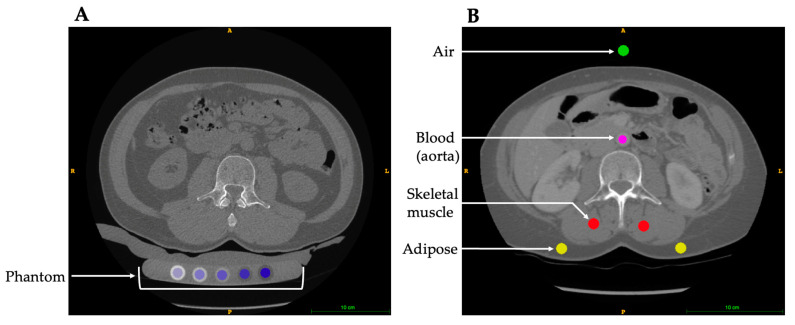
Abdominal axial cross-section at the level of the L3 vertebra. (**A**) Synchronous phantom calibration, placing a phantom under the participant during the CT scan; artificial colors (blue) have been added to highlight the calibration phantom rods. (**B**) Internal density calibration using referent tissues for calibration; artificial colors have been added to highlight the internal referent selections including air (green), adipose (yellow), blood (magenta), and skeletal muscle (red).

The basis of vBMD analysis using CT technologies relies on the conversion of HU, which provides a calibrated X-ray attenuation value defined by the densities of air and water, to vBMD (mg/cm^3^). While HU and vBMD both provide information about the density of bone, they are not directly interchangeable across CT scans. Given that vBMD is less susceptible to variability in X-ray attenuation due to patient characteristics, scan parameters, and X-ray tube drift, vBMD is a more appropriate measure for comparing different CT scans. There are two methods for calculating CT-derived vBMD, (1) phantom-based calibration and (2) phantomless internal calibration (Figure 1). Phantom-based calibration is the gold standard method, which involves using phantoms that contain rods of known dipotassium phosphate or calcium hydroxyapatite densities to convert HU values to vBMD values. The linear equation relating HU and vBMD varies by the type of phantom, scanner, protocol, location within the scanner bore (e.g., centred, or off-center), and patient size [4,19,20]. Phantom-based density calibration can either be performed synchronously by including a density calibration phantom with the patient within the scan field of view, or asynchronously by scanning the density phantom utilizing the same scanner and scan protocol but without the patient present [21,22]. Synchronous phantom calibration is the standard in the research setting and the resulting calibration equation is scan-specific. Asynchronous calibration is an alternative that requires relatively less time and resources as the phantom is scanned separate from the patient (e.g., daily or weekly), although the calibration equation is not scan-specific. Asynchronous calibration equations are reasonably accurate provided the X-ray tube is stable [22]; however, the calibration equations are specific to the scan protocol and phantom positioning for which they were acquired, and they cannot be employed in a retrospective study. A further limitation of this calibration technique is patient size: a smaller person compared to a larger person would have different beam-hardening artefacts; therefore, asynchronous calibration is limited in its accuracy.

Secondary analysis of clinically acquired CT scans for research purposes is limited by the fact that calibration phantoms are not typically included because they are not required for clinical diagnosis and increase scan time (e.g., additional setup, positioning, and image acquisition) as well as cost (e.g., phantom procurement and maintenance). As a result, research conducting analyses on clinically acquired CT scans often report native HU as an estimate of vBMD. Several studies have evaluated the application of HU thresholds for osteoporosis screening [9,11,23,24,25,26,27,28,29,30,31,32,33,34]; however, this method lacks reliability as HU are sensitive to X-ray energy and flux, beam hardening, positioning within the scanner, as well as different scanner makes and models [4,19,35,36].

Phantomless internal calibration is an alternative [6,37] to traditional phantom calibration that does not require scanning a phantom. It was first introduced using a histogram and peak fitting method developed in the late 1980s to quantify vBMD without the use of a calibration phantom [38,39]. This method quantified the frequency of HU within internal reference regions for skeletal muscle and adipose, resulting in two distinct peaks. Based on the known linear attenuation of these tissues, the peak HU values are extrapolated to create a standard calibration curve for the conversion of trabecular HU to vBMD. Although this technique is strongly correlated with phantom-based vBMD analysis, its utility has only been tested on specific CT scanners [38,39].

Building from this histogram method, two novel internal calibration methods have been developed [5,40,41]. The two methods both utilize air and several tissues (e.g., blood, adipose, cortical bone, and muscle) captured within the scan field of view as internal referents to convert HU to vBMD. However, these methods differ in the specific internal referents used and their procedure of HU to vBMD conversion. The first method involves correlating the native HU for air and blood or air and adipose internal referents with previously determined reference vBMD density values, deriving a scan-specific equation to convert HU to vBMD. The reference vBMD density values for each internal referent is determined using phantom calibrated scans from a cohort of patients [42]. There are limitations as this method depends on existing variation within the patient cohort used to determine the reference vBMD density values. The second method involves estimating the effective scan energy by iteratively correlating the HU and corresponding mass attenuation of air, blood, adipose, cortical bone, and muscle internal referents. The linear equation relating vBMD and HU is then mathematically derived [5].

Phantomless internal calibration provides accurate scanner-specific vBMD analysis when compared with traditional phantom-based calibration [5,6,37] and consistent inter-operator precision [6]. More specifically, the internal density vBMD calibration has a strong correlation with synchronous phantom density calibration at both the vertebra (R^2^ = 0.97–0.99) [5,6,37] and hip (R^2^ = 0.89–0.98) [5,6]. Previous research has tested combinations of internal referents to determine the lowest error using the phantomless internal calibration method [40]. There is no consensus on the combination of internal tissues to be used for the calibration, and these may be different whether the target analysis is skeletal muscle or bone. While skeletal muscle was previously included as an internal referent [5,43], recent findings discourage the inclusion of skeletal muscle as fatty infiltration is common with aging and results in altering composition [40]. The inclusion of cortical bone as an internal density referent remains to be debated, in part due to the density of cortical bone being much higher than the other internal referent tissues (e.g., adipose, blood, skeletal muscle) that are more similar on the calibration curve. A small shift in bone HU has a large influence on the line of best fit for the linear regression, and therefore influences the conversion of HU to vBMD that is being extrapolated. Conversely, a linear equation not including bone as an internal referent would be sensitive to any errors in the other internal referent tissues due to the extrapolation of the calibration curve. By including cortical bone, this accounts for a wide range of densities encompassed within the vBMD measurement. As for the other density referents, air should always be selected since it is independent of alterations in patient tissue composition due to pathologies, aging, and other factors [40]. Aside from air, the most selected tissues are blood and/or adipose tissue as these tissue types have relatively low inter-subject variation [40,41]. However, blood as an internal referent is not appropriate in contrast-enhanced scans.

## 3. Contrast Enhancement

Clinically acquired CT scans commonly use contrast agents to examine the vasculature, intestines, or other structures, for conditions such as hematuria or pulmonary embolisms. However, contrast alters the attenuation of tissues thereby limiting tissue selection for the phantomless internal calibration. Contrast agents, such as iodine-based and barium sulfate, are often used in the clinical setting to enhance CT image contrast by altering the X-ray attenuation of the vasculature or gastrointestinal track, respectively. This contrast enhancement also influences bone and muscle due to the contrast perfused blood vessels in and around these tissues. Anatomical sites surrounded by more vasculature (e.g., vertebra) are more influenced by the contrast compared to less vascularized more distal skeletal sites (e.g., proximal femur) [44]. Contrast enhancement increases lumbar vertebra HU compared to unenhanced CT scans by approximately 8–10% [45,46,47]. As density calibration is not able to fully account for this increase in bone X-ray attenuation, this bias remains after HU to vBMD conversion. Contrast enhancement increases synchronous phantom-derived vBMD values approximately 31% in the lumbar spine and 2% in the proximal femur compared to unenhanced CT scans. These variations can be attributed differences in vasculature and blood supply between these two skeletal sites [44]. It is worth noting the influence of contrast enhancement may differ among individuals with varying lumbar vBMDs. For instance, individuals with osteoporosis or osteopenia exhibit decreased vertebral marrow blood perfusion compared to individuals with a normal lumbar vertebra vBMD [48], further complicating the ability to account for contrast enhancement. Previous studies have demonstrated that accounting for contrast enhancement using corrections can similarly predict osteoporosis compared to unenhanced scans [44,45,46,49]. However, the bias associated with contrast-enhanced CT HU and phantom-derived vBMD may lead to inaccurate results for between group comparisons, multi-centre, or longitudinal studies.

To date there is limited research examining the influence of intravenous contrast in relation to internal calibration for opportunistic CT analysis. One study comparing calibration methods reported that internally calibrated contrast-enhanced CT scans overestimate vBMD, similarly to the observed overestimation of both synchronous and asynchronous phantom-derived vBMD values [50]. A challenge with contrast-enhanced CT analysis using internal calibration is the variable uptake of the administered contrast agents in the internal referent tissues due to differences in contrast perfusion. This is largely influenced by the time between the contrast administration and CT scan acquisition. Given that internal density calibration relies on the conversion of HU from various tissues to calibrate for bone density, it is important to choose appropriate tissues and correct for the influence of contrast agents to obtain accurate density measures [50]. Consideration of the differences in internal density techniques for calibration should also be accounted. Specifically, linear correction equations can be applied to correct for this bias [50], but further investigation is warranted to test the robustness of this correction across different scanners and protocols.

Other considerations include the delay between contrast administration and the CT scan (arterial vs. portal phase), and the amount of contrast agent administered, as these factors also impact tissue X-ray attenuation [47,51,52]. Standardizing correction methods for both contrast and contrast phase are particularly important for longitudinal studies to obtain accurate results [51,53]. Most recently, artificial intelligence (AI) has been developed to assess CT scans to identify contrast phase, [53,54].

## 4. Bone Segmentation

Coupled with calibration, accurate bone segmentation methods are essential for producing quantitative vBMD values. Currently, there are several methods being explored for segmenting bone captured in clinical CT scans. The high X-ray attenuation of bone in CT scans allows for semi-automated and AI to segment and identify skeletal sites [55,56,57,58,59,60]. Within the pipeline of CT bone analysis, bone segmentation is time consuming due to the required manual input and/or computing power. There are many commonly used semi-automated methods for bone segmentation, which include thresholding, region growing, and edge detection [61]. Common open-source programs used for semi-automated bone segmentation include 3D Slicer (http://www.slicer.org) [62], ITK-SNAP (http://www.itksnap.org) [63], and InVesalius (https://invesalius.github.io) [64,65], while commercially available software, such as Mindways, can be used for BMD analysis (Mindways Software Inc., Austin, TX, USA) or services, such as VirtuOst (O.N. Diagnostics, Berkley, CA, USA). However, there are limitations to these semi-automated segmentation methods such as (1) the required user input time to define hard constraints for the foreground and background, and (2) users must visually inspect and manually correct segmentation errors produced by these algorithms, which (3) reduces the reproducibility and introduces operator error [61,63,66]. Additionally, with the increase in the number of CT scans and the computational power required for the larger file sizes associated with high-resolution scans, this has rendered common image processing and manual segmentation methods to be unsuitable to effectively evaluate clinical CT scans for secondary musculoskeletal outcomes.

Although recent advances utilizing AI for automated bone segmentation [53,55,57] offer a more streamlined process, in some cases it may be difficult to automate, particularly in aging bones with poorly defined borders or abnormalities, as well as clinical scans reconstructed with coarse image resolution (e.g., 2.5 to 5 mm slice thickness in the axial plane). Slice thickness and in-plane pixel size significantly influences the accuracy of bone segmentations and vBMD measurements due to partial volume effects. Lower resolution CT scans reduce vBMD accuracy and trabecular bone is more affected than cortical bone due to the thin structures [67]. Although partial volume effects are unavoidable with CT imaging, the higher resolution improves vBMD accuracy. Anecdotally, a key challenge of repurposing clinical CT scans is the variance in scan resolution. Often, clinical CT scans are saved at a lower resolution for data storage purposes despite the original acquisition being at a high resolution.

Applying AI for bone segmentation and identification includes machine learning based on neural networks and deep learning. These methods require large training datasets that include “normal” and “abnormal” images for the development of a robust system to accurately identify bones. Machine learning methods are of particular interest as these methods can convert raw inputs to desired outputs by learning features from data using supervised training with CT datasets [66]. The U-net convolutional network is currently the most used machine learning approach applied to segmentation and requires fewer images for end-to-end training [68]. Of particular interest is segmenting the vertebra, in part due to the number of clinical chest, abdominal, and pelvic CT scans that capture the vertebra, and given that vertebral vBMD measures are a standard skeletal site for osteoporosis diagnosis [69].

The number of vertebra included in the CT scan field of view influences the accuracy of the vertebral identification [58]. Most clinical CT scans of the chest or abdomen do not capture the entire spine, or in some cases may not include the full vertebra of interest if the vertebra is located close to the edge of the scan field of view. Therefore, it is recommended that machine-learning-based identification and segmentation methods do not rely on certain vertebra or the number of vertebrae to be present within the scan to improve the utility of these algorithms [57]. Notably, transitional vertebrae are difficult to identify and segment due to their low clinical prevalence with an estimated 11% for T13 and 8% for L6 vertebra [70]. The thoracolumbar transitional T13 vertebra is often correlated with an abnormal rib count [70], complicating the correct vertebra identification using machine learning. An appropriate training set that mimics the prevalence of these transitional vertebrae is important for the development of robust machine learning models. Although vBMD has been demonstrated to decrease from the thoracic to lumbar spine, comparisons can still be drawn across skeletal sites since there is a strong correlation between regional skeletal sites [71,72].

As computational methods continue to advance, postprocessing will become more streamlined and standardized for the secondary analysis of bone. Machine learning is a promising method for broadly implementing segmentation and identification for CT scans. A key challenge will include standardizing these approaches to create a robust analysis that can be applied to a variety of different clinical CT scans.

## 5. CT Hardware Advances

Advances in CT hardware and computational developments have significantly improved CT imaging, enabling less radiation exposure, improved image resolution, faster scan times, and faster image reconstructions. An early major improvement was the progression from slice-by-slice scanning to the spiral CT scanners we use today, allowing for continuous scanning of the subject with faster scan times [73]. Modification to increase the number of detectors for multislice scanning over the past 30 years has significantly improved image quality as detector arrays have increased from 4 to 8, 16, 32, 40, and 64 slices, and now offer up to 320 slices per gantry rotation [73,74]. However, as CT technologies continue to advance, this also creates new challenges. A key challenge of opportunistic CT analysis is the lack of access to the clinically obtained raw data and control over the acquisition or reconstruction parameters. The secondary analysis of bone from clinical CT scans was first developed from standard multi-detector CT scanners but will continue to evolve as new technologies are developed to include cone beam CT (CBCT), dual energy CT (DECT), and photon counting CT (PCCT).

As the name suggests, CBCT emits a cone-shaped X-ray beam instead of the conventional fan beam. Due to CBCT’s lower radiation dose, this technique is common practice in the dental setting to evaluate the jaw, dentition, and bony structures of the head [75]. Although previous research has demonstrated that individuals with osteoporosis also have lower mandibular vBMD measured using CBCT [76,77,78], mandibular vBMD is not clinically used to diagnose osteoporosis. More recently, CBCT has been marketed for orthopaedic settings to provide accurate vBMD measurements and improve surgical planning. However, there are concerns about the inhomogeneity of vBMD measurements and geometric distortions through the field of view due to the wide cone angle. The reliability of HU from CBCT scans is affected by several factors. In particular, CBCT are more prone to X-ray beam-hardening artefacts and radiation scattering effects associated with the cone shape of the X-ray beam [79,80,81]. This can result in inconsistent HU values within the same anatomical structure or across different CBCT scans [82]. Compounding this HU inconsistency is the lack of standardized calibration phantoms used in CBCT systems. Despite these challenges, recent advancements in CBCT technology, such as improved calibration methods and image reconstruction algorithms, have aimed to mitigate some of the reliability issues [79,83].

Another key challenge of CT imaging analysis is that tissues of different chemical compositions can have similar X-ray attenuations, making it difficult to classify tissues. Unlike clinical single-energy CT scanners that use polychromatic X-ray energies, DECT uses two polychromatic X-ray energies. DECT takes advantage of tissues’ X-ray attenuation coefficient differential change across X-ray spectrums with different kVp. Hardware advances have improved tissue differentiation either using two energies (source-based) or a detector that separates signals by resolving different energies (detector-based) [84,85]. Postprocessing material decomposition is particularly useful for mapping and removing substances of known attenuation characteristics such as contrast agents (e.g., iodine) typically used in the clinical setting to create virtual non-contrast images [85,86]. More recently, DECT phantomless calibration has shown promise for estimating vBMD [87,88,89,90], demonstrating strong agreement with DXA aBMD measures [87,88]. However, best practices for integrating internal density calibration with DECT have yet to be determined.

Most recently, PCCT has the potential to dramatically change CT imaging, with improved spectral sensitivity, better spatial resolution, reduced noise, fewer image artifacts, and lower radiation doses [91,92]. PCCT detectors measure both the number of photons and sorts them into bins according to their X-ray energy, enabling material decomposition [93,94]. Unlike DECT, PCCT has the ability to derive multi-energy images through a single polychromatic X-ray energy scan [95]. This allows greater sensitivity to absorption edges, and therefore, contrast-enhanced PCCT scans require less contrast agent or radiation exposure to obtain iodine contrast visibility [95]. PCCT is a promising new technology to revolutionize medical imaging. The development of these new hardware advances provides exciting new opportunities to leverage clinical imaging data.

## 6. Future Directions

In summary, opportunistic CT analysis allows for the secondary analysis of bone using the plethora of retrospective clinical CT data, providing a remarkable opportunity to repurpose previously acquired clinical data. To further enhance the robustness of this technique, it is crucial to continue optimizing opportunistic CT bone analysis. An essential aspect is the internal calibration, which allows for accurate comparisons across multiple sites and scanners. Notably, internal calibration has already demonstrated excellent performance in single-energy CT scanners. By integrating internal calibration with emerging technologies such as DECT and PCCT, we can harness the potential of these advanced imaging techniques to further improve the analysis. Given the predictability of tissue interactions with X-rays, the transition to combining internal calibration with these imaging modalities may not require significant leaps in methodology. Although further research, development, and advancements in computation and hardware are necessary, the synergistic combination of these techniques holds immense promise for unlocking new opportunities in data analysis. By capitalizing on these advancements, we can overcome existing hurdles and continually enhance the analysis using the opportunistic CT technique. Aside from research purposes, it is important to develop and integrate the opportunistic CT technique into the clinical setting for osteoporosis screening. Given the inherent variations in healthcare systems, the incorporation of opportunistic CT analysis into clinical workflows will pose additional challenges including extra training for healthcare professionals and more standardized CT image collection and recording. In conclusion, opportunistic CT analysis is a remarkable approach and the integration of internal calibration with emerging imaging technologies has the potential to revolutionize the field. While challenges exist, ongoing research, technological advancements, and computational progress will undoubtedly lead to improved analysis and open new avenues for exploration.

## Data Availability

Not applicable.
